# Welcoming new leadership team of the Plant Biotechnology Journal

**DOI:** 10.1111/pbi.13988

**Published:** 2023-01-09

**Authors:** Henry Daniell

**Affiliations:** ^1^ W.D. Miller Professor and Director of Translational Research, School of Dental Medicine University of Pennsylvania Philadelphia Pennsylvania USA

Welcome to this first issue of the 21st volume of the *Plant Biotechnology Journal* (PBJ), an open access plant science journal offering free global access to our readers through the open access fee paid by our authors. In this editorial, I introduce the new Editor‐in‐Chief, Prof. Johnathan Napier, the Executive Editor, Prof. Shangxia Jin, and Senior Editors Profs. Dominique Michaud and Rajeev Varshney as I hand over responsibility of PBJ Editorin‐Chief. Going forward, all of them will make final decisions on manuscripts submitted to PBJ.

**Figure 1:**
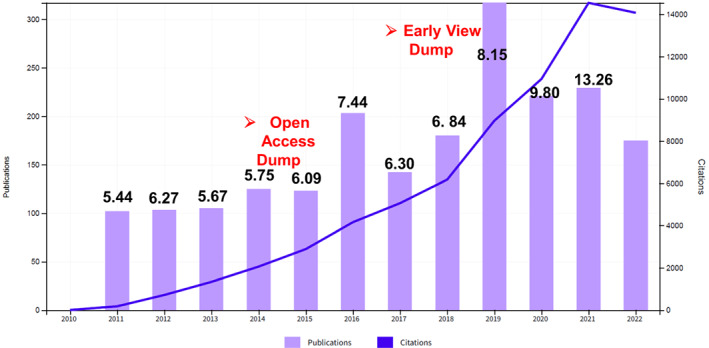
Publications and citations of the Plant Biotechnology Journal since 2011. Excluding Brief Communications, the number of articles published in PBJ increased from 102 in 2011 to 229 in 2021. The impact factor during this period increased from 5.44 to 13.26 and total citations increased from 715 to 14,541. There are two anomalies in the number of published articles due to the open access transition in 2016 and Web of Science inclusion of Early View articles in 2019.

Over the last 10 years, PBJ has grown steadily in the number of articles published, from ~102 articles in 2011 to ~229 articles (excluding Brief Communications) in 2021, with 715 citations in 2012 to 14 541 in 2021. During this period, PBJ's impact factor (IF) has increased steadily from 5.44 in 2011 to 13.26 in 2021 (Figure 1). Irrespective of the evaluation metrics used (IF or CiteScore), PBJ currently ranks third among plant science journals publishing original research, and Scopus CiteScore continues to rank PBJ first among 370 Agronomy and Crop Science journals.

These accomplishments would not have been possible without the contributions of their best research by our scholarly authors and dedicated service of our expert reviewers and editors. I would like to acknowledge service of all Associate Editors who served in the past 10 years of my tenure as the Editor in Chief, including Drs. Robert Henry (2002–2014), Robert Birch (2002–2013), Loïc Faye (2002–2012), Paul Quick (2002–2012), Dominique Michaud (2007‐present), Malcolm Campbell (2008–2021), Xiao‐Ya Chen (2013‐present), Rajeev Varshney (2013‐present), Qifa Zhang (2013–2016), Johnathan Napier (2014‐present), Joseph Petolino (2014–2016), Dave Edwards (2011–2021), Neal Stewart (2012‐present), Stephen Streatfield (2012‐present), Davies Maelor (2010–2014), Martin Parry (2016‐present), Nicola Patron (2016‐present), Daoxiu Zhou (2016–2019), Zuhua He (2017‐present), Kan Wang (2017‐present), Shuangxia Jin (2018‐present), Jihong Liu‐Clarke (2018–2020), Marco Maccaferri (2018–2021), Xuehui Huang (2020‐present), Nils Stein (2020‐present), Yanfei Mao (2020‐present), Bing Yang (2020‐present), Thomas Jacobs (2020‐present), Leena Tripathi (2020‐present), Jacqueline Batley (2021‐present), Francois Belzile (2018‐present), Caixia Gao (2019–2020), Anthony Hall (2020–2021), Yiping Qi (2020‐present), Mario Caccamo (2021‐present), Nigel Halford, (2021‐present) and Wolfram Weckwerth (2021‐present). Prof. Dominique Michaud started serving as the Senior Editor 3 years ago and Profs. Shuangxia Jin, Johnathan Napier, Rajeev Varshney served as Senior Editors since last year.

Despite COVID‐19 pandemic, the number of submissions to PBJ has continued to increase in 2022. PBJ has received manuscripts from 46 countries in 2022, representing all continents around the globe, highlighting the breadth of countries that submit to PBJ. Readership is also increasing rapidly and readers from over 215 countries have downloaded papers publishing in PBJ in 2022. We are on track to have over 1.5 million full text downloads of PBJ articles in 2022.

PBJ has significantly increased social media activities in 2022, with the help of Prof. Shuangxia Jin (PBJ Senior Editor, Huazhong Agricultural University, China) launching the PBJ WeChat account (PBJ ID: PBJ201903) on 1 March 2019. In 2022, this WeChat account has published 1370 news articles including 343 original articles written by his students and 1027 articles cited from other social media sources. This has resulted in 69 000 followers, with 3 218 675 hits from 1 335 352 computers, 11 125 hits per day and 2930 hits per news of each publication from *Plant Biotechnology Journal*. PBJ WeChat account has been recognized in the Top 10 academic WeChat accounts in China since 2020, along with Cell Press, Springer/Nature, Science/AAAS, NEJM, The Lancet, Elsevier, ACS and RSC. Wiley Plant Science tweets @wileyplantsci, now has >20 500 followers and publications of global interest are shared through this.

Without the outstanding leadership of Ms. Rosie Trice, Senior Publishing Manager at Wiley, Oxford, PBJ would not be able to function and I convey my deepest appreciation, especially on expanding the editorial board. I thank PBJ production editor at Wiley, Ms. Rajalakshmi Sundararamanujam for production from Chennai, India and timely production and release of PBJ issues every month, Ms. Madhura Amdekar for her expertise in evaluation of image manipulation, Ms. Reshma Raghu for editorial handling of manuscripts and Ms Andrea Lewis for the overseeing the PBJ Editorial Office.

In 2016, PBJ transitioned from a subscription printed journal to an open access online journal, offering open access for all articles published since inception. PBJ continues to grow by publishing more articles and improving ranking among the plant science or biotechnology journals, without any negative impact on transition to open access. However, the cost of open access fee has been transferred from libraries to corresponding authors and this, unfortunately, has negatively impacted investigators with limited funding. My sincere apologies for authors who are unable to publish in PBJ due to such financial challenges.

As I reflect the past 20 years of my association with PBJ, I am truly thankful to Prof. Keith Edwards for inviting me to join the journal as a founding editor and then persuading me to take on the role of Editor‐in‐Chief in 2011, with the strong support of Prof. Paul Hutchison at PBJ management. I thank all the Associate and Senior Editors listed above for their dedication and friendship throughout this period. I wish PBJ continued success. I will no longer handle PBJ manuscripts, but will continue to offer guidance to the new PBJ team, serving as the Editor Emeritus.

